# Supplementary material for: Retinal Organoids from an AIPL1 CRISPR/Cas9 Knockout Cell Line Successfully Recapitulate the Molecular Features of LCA4 Disease
Source: Int J Mol Sci. 2023 Mar 21;24(6):5912. doi: 10.3390/ijms24065912 (PMC10057647; doi:10.3390/ijms24065912)
Supplement: Supplementary file 1 [file ijms-24-05912-s001.zip › ijms-2223590-supplementary.pdf]

Supplementary information: Supplementary Figures:

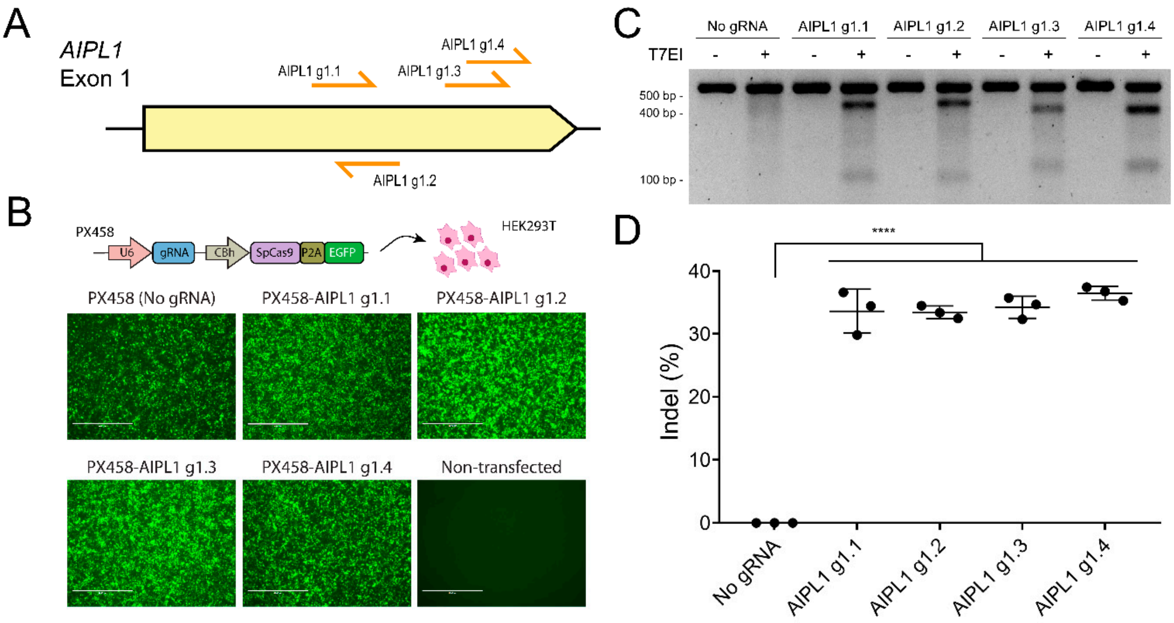

Figure S1: Design of CRISPR/Cas9 sequences for targeted *AIPL1* gene knockout. **(A)** Schematic illustration of guide RNAs (gRNA) designed to direct the CRISPR/Cas9 endonuclease to *AIPL1* exon 1. **(B) (Top)** Schematic illustration of CRISPR/Cas9-encoding vector PX458 for transfection into human embryonic kidney 293T (HEK293T) cells. Targeting gRNA is driven by the U6 promoter, while *Streptococcus pyogenes* Cas9 nuclease (SpCas9) and a EGFP reporter separated by a T2A cleavage peptide are driven by the CBh promoter. **(Bottom)** Fluorescence GFP imaging of transfected HEK293T cells show high levels of PX458 transfection similar across all transfected constructs. Scale bar, 1000 µm. **(C)** Analysis of CRISPR/Cas9-mediated targeted indel formation by T7 Endonuclease I (T7EI) assay. Fragmented bands of a lower molecular weight result from the indel formation and demonstrates that cells transfected with *AIPL1*-targeting PX458 constructs (AIPL1 g1.1-4) were able to promote double-strand breaks in their respective target sequences. **(D)** Quantification of indel formation by densiometric analysis of T7EI assay indicates a level of 30-35% cutting efficiency across all targeting *AIPL1* gRNAs. Data expressed as Mean ± SD (N=3). **** $p<0.0001$, Student's t test.

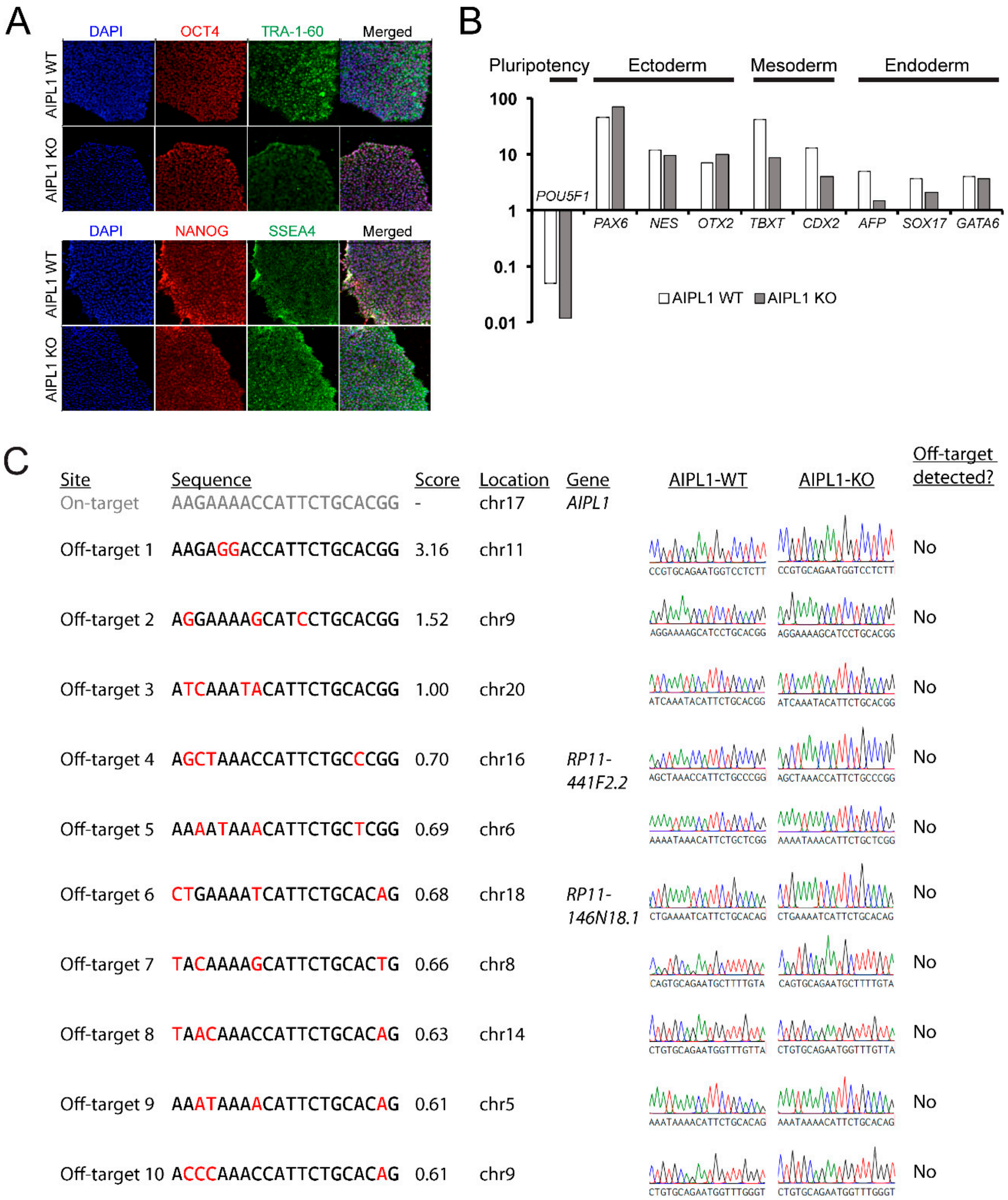

Figure S2: Characterization of AIPL1 isogenic iPSC clones. **(A)** Immunocytochemistry analysis of pluripotency markers Oct4, Tra-1-60, Nanog and SSEA4 markers in AIPL1-WT and AIPL1-KO isogenic iPSC lines. Scale bar = 10 µm. **(B)** Trilineage differentiation potential of AIPL1-WT and AIPL1-KO isogenic iPSC lines into ectoderm, endoderm and mesoderm germ layers was confirmed using gene markers of differentiation. **(C)** Sanger sequencing analysis of CRISPR/Cas9 off-target mutations in AIPL1-WT and AIPL1-KO isogenic iPSC lines. Top 10 off-target sequences predicted *in silico* with highest homology to AIPL1 g1.4 (On-target) are illustrated, along with predicted off-target score, chromosomal location and targeted gene. Mismatches to the on-target sequences are highlighted in red.

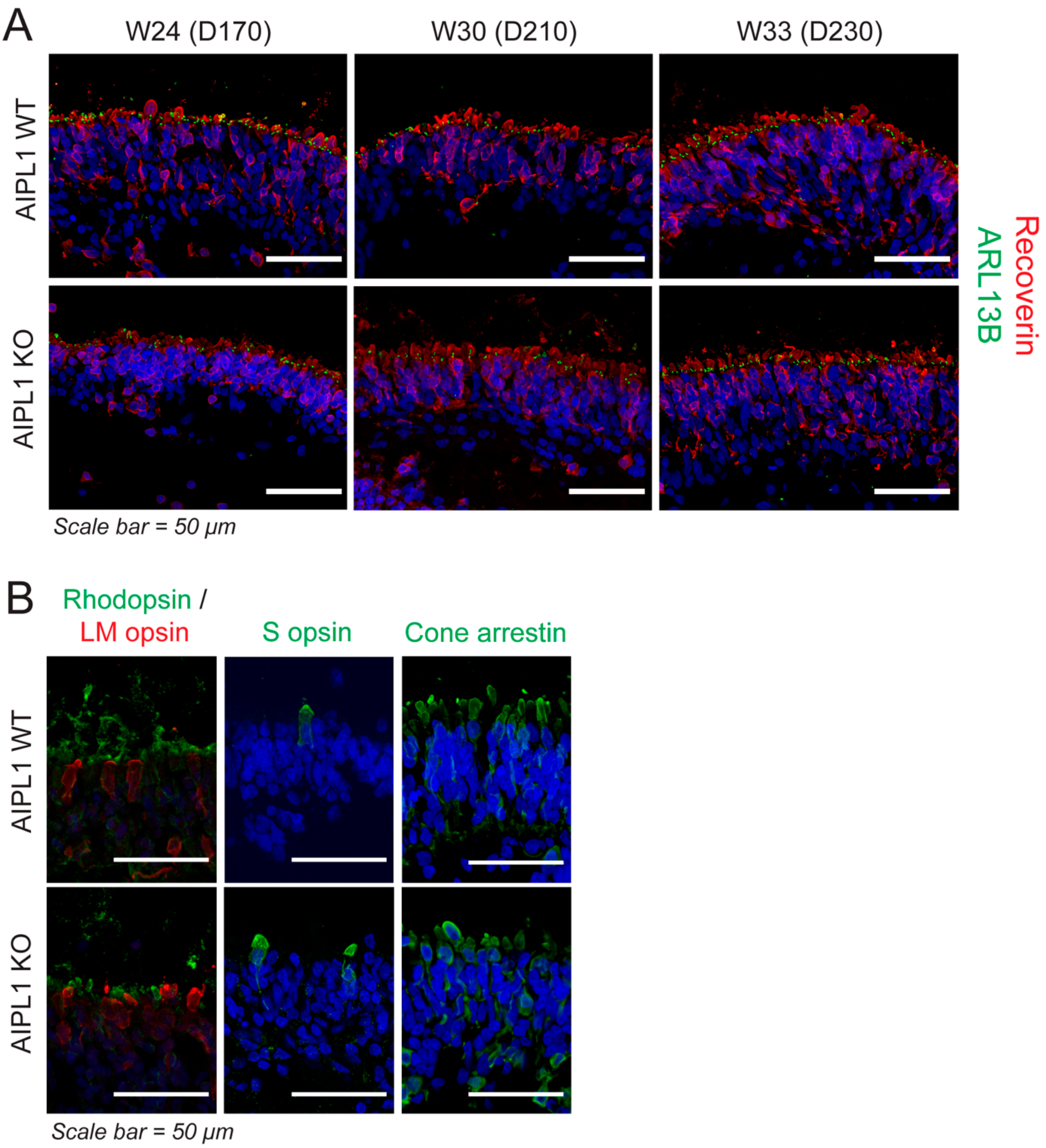

Figure S3: Immunocytochemistry staining of additional photoreceptor markers in AIPL1 isogenic retinal organoids. **(A)** The photoreceptor marker recoverin (red) and photoreceptor cilia marker ARL13B (green) were investigated in AIPL1-WT and AIPL1-KO retinal organoids at day (D) 170, D210 and D230. Ubiquitous distribution of recoverin was observed in the outer nuclear layer while ARL13B was detected in photoreceptor cilia, both as expected. **(B)** Rod (rhodopsin) and cone (LM opsin, S opsin and cone arrestin) markers were investigated in D230 AIPL1-WT and AIPL1-KO retinal organoids. Consistent abundance of rods (rhodopsin), cones (cone arrestin), as well as red/green cones (LM opsin) and blue cones (S opsin) were detected within the outer nuclear layer of AIPL1-WT and AIPL1-KO retinal organoids. Scale bar = 50 µm.

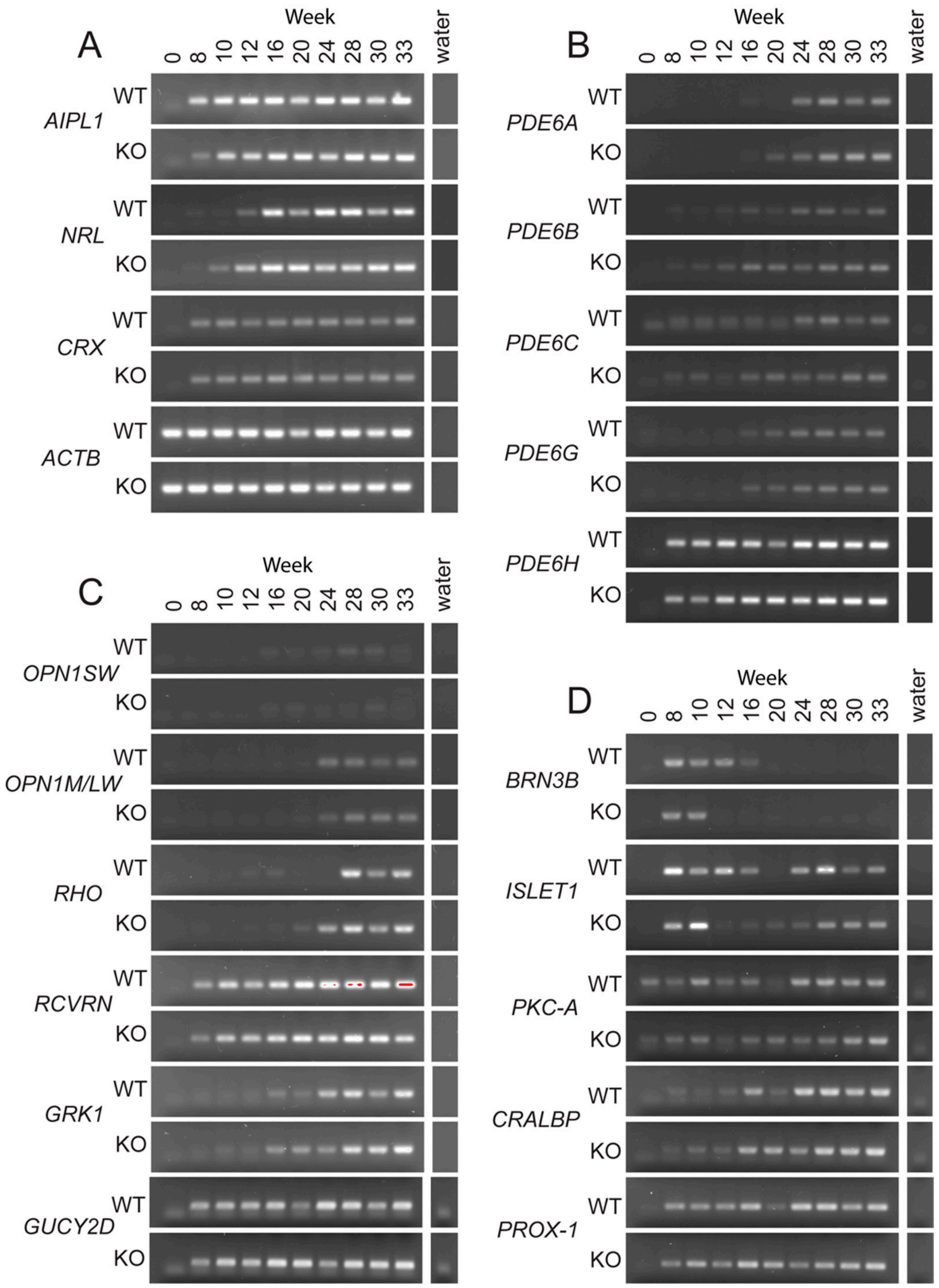

Figure S4: Temporal gene expression of relevant retinal markers in AIPL1 isogenic retinal organoids; week 0 = expression in iPSC prior to the start of differentiation. RT-PCR was used to assess the temporal gene expression for a range of markers, specifically **(A)** *AIPL1*, photoreceptor-specific transcription factors (*NRL, CRX*) and the β-actin gene (*ACTB)* as a loading control; **(B)** *PDE6* in rod (*PDE6A, PDE6B, PDE6G*) and cone (*PDE6C, PDE6H*) photoreceptors; **(C)** photoreceptor-specific markers in photoreceptors (*RCVRN, GRK1, GUCY2D*), rods (*RHO*) and cones (*OPN1SW, OPN1M/LW*); and **(D)** markers of other non-photoreceptor cell types typically found within the retina (retinal ganglion cells (*BRN3B, ISL1*), bipolar cells (*PKC-A*), Müller glia (*CRALBP*) and amacrine and horizonal cells (*PROX1*).The temporal gene expression patterns were similar in AIPL1-WT and AIPL1-KO RO.

Supplementary Information: Supplementary Tables

Table S1. Top AIPL1-targeting guide RNA sequences selected by *in silico* analysis.

Name	Sequence (5'-3')	On-target score[a]	Off-target score[b]
AIPL1 g1.1	TGCCGCTCTGCTCCTGAAC	61.1	41.9
AIPL1 g1.2	TTCCACGTTCAGGAGCAGAG	74.1	37.4
AIPL1 g1.3	CAAGAAAACCATTCTGCACG	61.6	41.9
AIPL1 g1.4	AAGAAAACCATTCTGCACGG	71.2	42.5

[a]Predicted by Benchling (https://www.benchling.com/). Score illustrated from 0-100 and directly correlated to predicted cutting efficiency.
[b]Predicted by Benchling (https://www.benchling.com/). Score illustrated from 0-100 and directly correlated to predicted target specificity.

Table S2. Primers for AIPL1 gRNA used for cloning AIPL1 gRNAs and for amplification of AIPL1 exon 1 for T7EI assay.

Name	Sequence (5'-3')
AIPL1 g1.1-F	caccgTGCCGCTCTGCTCCTGAACG
AIPL1 g1.1-R	aaacCGTTCAGGAGCAGAGCGGCAc
AIPL1 g1.2-F	caccgTTCCACGTTCAGGAGCAGAG
AIPL1 g1.2-R	aaacCTCTGCTCCTGAACGTGGAAc
AIPL1 g1.3-F	caccgCAAGAAAACCATTCTGCACG
AIPL1 g1.3-R	aaacCGTGCAGAATGGTTTTCTTGc
AIPL1 g1.4-F	caccgAAGAAAACCATTCTGCACGG
AIPL1 g1.4-R	aaacCCGTGCAGAATGGTTTTCTTc
AIPL1 Exon1-F	GCTGGGTAAATCCCAGAGTCTCAGC
AIPL1 Exon1-2R	AAAGCCCGAATTCTGTAGCCACAGA

Table S3. Primary and secondary antibodies used in immunofluorescence (IF) and western blot (WB) analysis.

Antibody	Species	Fluorophore conjugate	Supplier	Cat number	Dilution
AIPL1	Rabbit	-	Produced in-house	N/A	1:250 (IF)
AIPL1	Rabbit	-	V. Ramamurthy lab	N/A	1:1000 (WB)
ARL13b	Mouse	-	Neuromab	75-287	1:1000 (IF)
Cone arrestin	Mouse	-	Millipore	MABN2636	1:250 (IF)
GAPDH	Mouse	-	ProteinTech	60004-1-Ig	1:10,000 (WB)
L/M Opsin	Rabbit	-	Millipore	AB5405	1:500 (IF)
NANOG	Rabbit	-	Abcam	AB21624	1:200 (IF)
OCT4	Rabbit	-	Abcam	AB19857	1:1000 (IF)
PDE6A	Rabbit	-	ProteinTech	21200-1-AP	1:1000 (IF) 1:3000 (WB)
PDE6B	Rabbit	-	Thermo Fisher Scientific	PA1-722	1:500 (IF)
Rhodopsin	Mouse	-	Millipore	MABN15	1:1000 (IF)
Recoverin	Rabbit	-	Abcam	Ab5585	1:1000 (IF + WB)
S Opsin	Rabbit	-	Millipore	AB5407	1:200 (IF)
SSEA4	Mouse	-	Cell Signalling	MC813	1:300 (IF)
TRA-1-60	Mouse	-	Cell Signalling	4746	1:500 (IF)
α-mouse IgG	Donkey	AF488	Thermo Fisher Scientific	A-21202	1:1000 (IF)
α-mouse IgG	Donkey	AF555	Thermo Fisher Scientific	A-32773	1:1000 (IF)
α-mouse IgG	Donkey	AF647	Thermo Fisher Scientific	A-31571	1:1000 (IF)
α-rabbit IgG	Donkey	AF488	Thermo Fisher Scientific	A-21206	1:1000 (IF)
α-rabbit IgG	Donkey	AF555	Thermo Fisher Scientific	A-31572	1:1000 (IF)
α-rabbit IgG	Donkey	AF647	Thermo Fisher Scientific	A-31573	1:1000 (IF)
α-mouse IgG	Goat	HRP	Thermo Fisher Scientific	31430	1:10,000 (WB)
α-rabbit IgG	Goat	HRP	Thermo Fisher Scientific	31461	1:10,000 (WB)
Phalloidin	-	AF488	Invitrogen / Molecular probes	A12379	5 U/mL (IF)

Table S4. Primer sequences for RT-PCR analysis of retinal markers.

Marker	Forward	Reverse	Expected product size (bp)
ACTB	GCGAGAAGATGACCCAGATC	CCAGTGGTACGGCCAGAGG	103
AIPL1	ACCGGATCCCGAGTGATCTT	CGATGATGATGTGCATGGGC	66
BRN3B	CTCGCTCGAAGCCTACTTTG	GACGCGCACCACGTTTTTC	100
CRALBP	AAGCTGGCTACCCTGGTGT	TGAAGCAATATGCCTGCAAGA	126
CRX	TTTGCCAAGACCCAGTACC	GTTCTTGAACCAAACCTGAAC	96
ISLET1	GCGGAGTGTAATCAGTATTTGGA	GCATTTGATCCCGTACAACCT	102
NRL	CACTGACCACATCCTCTCGG	GAGGGTTCCCGCTTTACCTC	141
OPN1LW/MW	CCTATGTGTGTCCTGGAGG	CATCCATCTCTCCCAGGAAATG	90
OPN1SW	CATGTTTGTGCTTTGGAGG	CGAAGGGCTTACAGATGAC	109
PDE6A	TAACGTCCCCAACACAGAGG	CCACCACATCCTTCCCATTC	116
PDE6B	GACGTGTGGTCTGTGCTGAT	CTTGCCGTGGAGGATGTAGTC	111
PDE6C	GTCACTAAGAACCTGCTGGCAACC	AAAGACCTCTTCATCCTGTTTGG	117
PDE6G	AAGCAGCGACAGACCAGG	TGTGATGTCTGTTCCCAGGC	105
PDE6H	GAGGCAGACTCGCCAATTC	GTGGCTGAATGCCTCCCA	130
PKC-A	GTCCACAAGAGGTGCCATGAA	AAGGTGGGGCTTCCGTAAGT	122
PROX-1	TGAAGACCTACTTCTCCGAC	GACGTGCGTACTTCTCCATC	114
RCVRN	ATGAAGTGCTGGAGATCGTC	ATCTTCTCGGCTCGCTTTTC	106
RETGC	ACTGTCCCTCTGAAGGCAG	CGTCATAGATGGTGCCAAAG	117
RHO	ACCAGCACCCTCTACACCTCTC	AGGACCACAGGGCAATTTC	109
